# Increased Phosphorylation of Vimentin in Noninfiltrative Meningiomas

**DOI:** 10.1371/journal.pone.0009238

**Published:** 2010-02-16

**Authors:** Ali Bouamrani, Claire Ramus, Emmanuel Gay, Laurent Pelletier, Myriam Cubizolles, Sabine Brugière, Didier Wion, François Berger, Jean-Paul Issartel

**Affiliations:** 1 Grenoble Institut des Neurosciences, INSERM U836, Université Joseph Fourier, Grenoble, France; 2 Department of Neurosurgery and Pathology, Centre Hospitalier Universitaire, Grenoble, France; 3 Ciphergen Inc., Fremont, California, United States of America; 4 CEA, Grenoble, France; 5 Centre National de la Recherche Scientifique (CNRS), Grenoble, France; Griffith University, Australia

## Abstract

**Background:**

Tissue invasion or tissue infiltration are clinical behaviors of a poor-prognosis subset of meningiomas. We carried out proteomic analyses of tissue extracts to discover new markers to accurately distinguish between infiltrative and noninfiltrative meningiomas.

**Methodology/Principal Findings:**

Protein lysates of 64 different tissue samples (including two brain-invasive and 32 infiltrative tumors) were submitted to SELDI-TOF mass spectrometric analysis. Mass profiles were used to build up both unsupervised and supervised hierarchical clustering. One marker was found at high levels in noninvasive and noninfiltrative tumors and appeared to be a discriminative marker for clustering infiltrative and/or invasive meningiomas versus noninvasive meningiomas in two distinct subsets. Sensitivity and specificity were 86.7% and 100%, respectively. This marker was purified and identified as a multiphosphorylated form of vimentin, a cytoskeletal protein expressed in meningiomas.

**Conclusions/Significance:**

Specific forms of vimentin can be surrogate molecular indicators of the invasive/infiltrative phenotype in tumors.

## Introduction

Currently, typing and grading a newly diagnosed tumor, the first steps in appropriate treatment, mainly rely on pathology analysis and a few biological markers with unambiguous diagnostic values. There is a need for new diagnosis markers that will also provide insights into certain specific features of tumors, such as invasiveness, proliferation, or cytotoxic drug sensitivity. Accurate markers for early detection of tumors that should thus improve the efficacy of therapy are also awaited.

Valuable markers for tumor diagnosis can be discovered using high-throughput screening of proteins or peptides in biological samples, in particular using mass-spectrometry techniques. With this strategy, comparison of protein profiles from sera [1] or from tissue sections [2], [3] has been put forward as a diagnostic approach for tumor characterization. Although the clinical value of the protein profiles was questioned [4], individual, valuable markers have been discovered in sera and identified based on the surface-enhanced laser desorption/ionization time-of-flight mass spectrometry platform (SELDI-TOF mass spectrometry), for example [5], [6]. SELDI-TOF mass spectrometry is an attractive analytical technology that combines selective retention of proteins on miniaturized chromatographic surfaces with mass spectrometric analyses. This facilitates detection and further purification and identification of biomarkers [7].

The goal of this study was to identify biomarkers that could help to discriminate different subsets of meningiomas. This work specifically focused on the study of the infiltrative behavior of this type of tumor. Meningiomas are slow-growing, extra-axial, and usually histologically benign tumors. According to the WHO classification, mainly based on histological features, meningiomas are classified as Grade I (meningiomas with a low risk of aggressive growth), Grade II (atypical meningiomas), or Grade III (anaplastic meningiomas) [8]. Interestingly, even in their low-grade status, meningiomas may exhibit particular phenotypes that are infiltrative to adjacent tissues. One of these phenotypes is characterized by meningioma cell infiltration into adjacent brain tissue. Typically, this event is specifically called invasion. Brain invasion is known to be correlated with a high risk of recurrence and aggressive meningioma behavior [9]. In addition, other infiltrative events are characterized by individual or combined infiltrations of meningioma cells into the adjacent bone or the large “interdural” venous cavities of the skull, such as the cavernous sinus or the venous sinuses. Taken all together, infiltrative phenotypes and brain invasion can be observed in about 20% of cases, and makes surgery very difficult. The different infiltrative phenotypes are not strictly correlated to either severe proliferation or malignant transformation and it is generally agreed that the histological appearance of meningiomas often fails to accurately distinguish between benign meningiomas and potentially infiltrative or invasive ones, although MRI and CT imaging show evidence of tumoral infiltration. Molecular biology studies of these tumors may provide a better appraisal of the clinical and biological potentials of the tumors. In addition to their prognostic value, biological markers may also offer opportunities to develop new therapeutic strategies to prevent tumor progression. With regard to meningiomas, strategies based on the sex-steroid receptor and some available drugs such as mifepristone have been proven to be partially effective [10], but the validation of new therapeutic approaches requires additional study.

We looked for biomarkers directly in tissues by running a multi-protein detection study using the SELDI-TOF technology. We found specific discriminative proteomic profiles that help to distinguish the noninfiltrative and noninvasive meningiomas from the other infiltrative tumors. Moreover, we purified one phosphorylated form of vimentin acting as an accurate marker for specific and sensitive identification of noninvasive meningiomas.

## Materials and Methods

### Sample Origin

A total of 64 meningioma tissues were collected from surgery and immediately frozen and stored at −80°C.

Tumor features such as sinus or bone infiltrations were routinely detected on preoperative MR and CT images. Diagnosis and bone infiltration were ascertained by pathology examination on paraffin-embedded tissue samples. Cavernous sinus or venous sinus infiltrations were confirmed by pathology examination for the presence of meningioma cells inside adjacent tissues when it was possible to do so since pathology examinations were most often carried out on resected samples from outside the sinuses. Brain invasion was confidently ascertained by pathology examination.

Of the 64 samples, two tumors exhibited brain invasion (tumors from patients P1 and P24); 32 tumors showed infiltrative behavior (toward one or several vicinal tissues) (tumors from patients P2–P23 and P25–P34); the other tumors were considered noninfiltrative and noninvasive (tumors from patients P35–P64).

Tumors were classified using the 2007 WHO classification [8]. Brain-invasive tumors were classified as Grade II, as were tumors with a high number of mitoses per field as recommended by Perry et al. [11].

Vimentin, the usual marker for meningioma, was detected immunohistochemically according to a standard method [12]. A total of 64 patients' samples with pathological and clinical features, as reported in Table 1, were studied and allocated to two groups (for statistical analysis purposes; see below).

**Table 1:** Patient clinical data.

Patients	Age (years)	Gender	Location	Infiltrated tissues (on MRI and/or CT)	Infiltrated tissues (pathological examination)	Recurrence	WHO grade	Pathology subtype	MIB-1	Mitoses in 10 fields (40×)
P1	68	F	SB (spheno-orbital)	bone	bone and brain (**invasion**)	no	II	M	3<M<5%	
P2	44	F	convexity (P)	bone	bone	no	I			0
P3	61	F	SB (petrous bone)	bone	nd	no	I	M	<1%	0
P4	36	F	SB (spheno-orbital)	bone	bone	no	I	M		0
P5	49	F	convexity (P)	bone	bone	no	I	M		0
P6	66	F	convexity (T)	bone	bone	no	I	M	<3%	0
P7	49	F	convexity (F)	bone	bone	no	I	S		0
P8	49	F	SB (spheno-orbital)	bone	bone	no	I	T	<1%	0
P9	54	F	SB (spheno-orbital)	bone	bone	no	I	M		0
P10	68	F	convexity (P)	bone	bone	no	II*		<3%	4<M<20 *
P11	50	F	SB (spheno-orbital)	bone, cavernous sinus	bone	no	I	M	<1%	0
P12	55	M	parasagittal	bone, sup longitudinal sinus	bone	no	I	F		0
P13	54	F	parasagittal	bone, sup longitudinal sinus	veinous lumen	no	I	M		0
P14	68	F	parasagittal	bone, sup longitudinal sinus	Bone+veinous lumen	no	I	M	<3%	0
P15	68	F	SB (cavernous sinus)	bone, cavernous sinus	bone	yes	I	M		0
P16	55	F	SB (spheno-orbital)	bone, cavernous sinus	bone	yes	I	T		0
P17	55	F	SB (cavernous sinus)	bone, cavernous sinus	bone	yes	I	M		0
P18	64	F	SB (spheno-orbital)	bone, cavernous sinus	bone	no	I	M		0
P19	52	M	SB (cavernous sinus)	bone, cavernous sinus	nd	yes	I	M		<1
P20	66	F	SB (spheno-orbital)	bone, cavernous sinus	bone	yes	I	F		0
P21	45	F	SB (cavernous sinus)	bone, cavernous sinus	nd	yes	I	M		0
P22	41	F	SB (spheno-orbital)	bone, cavernous sinus	yes	yes	I	M		0
P23	61	F	SB (spheno-orbital)	bone, cavernous sinus	bone	no	I	M		0
P24	64	F	SB (clivus)	brain	brain (**invasion**)	yes	II	M		
P25	52	M	SB (cavernous sinus)	cavernous sinus, no bone	Nd	no	I	T	3%	0
P26	45	F	SB (cavernous sinus)	cavernous sinus, no bone	Nd	yes	I	M		0
P27	67	M	SB (cavernous sinus)	cavernous sinus, no bone	Nd	no	I	M		0
P28	45	F	SB (clinoid)	cavernous sinus, no bone	Nd	no	I	M	3%	0
P29	79	F	parasagittal	sup longitudinal sinus	veinous lumen	no	I	M		0
P30	71	F	convexity (PF)	vein (lateral sinus)	Nd	no	I	T		0
P31	55	F	parasagittal	vein (sup longitudinal sinus)	inside the veinous lumen	no	I	M		0
P32	55	F	SB (cavernous sinus)	cavernous sinus, no bone	Nd	no	I	M		0
P33	69	F	SB (cavernous sinus)	cavernous sinus, no bone	Nd	no	I	M		0
P34	73	F	convexity (PF)	lateral sinus	Nd	no	I	F		0
P35	43	F	parasagittal	no	no	no	I	T	<3%	0
P36	46	F	convexity (P)	no	No	no	I	M	<1%	0
P37	62	M	SB (frontal)	no	No	no	I	M	<1%	1
P38	71	F	convexity (T)	no	no	no	I	M	<1%	0
P39	56	F	SB (temporal)	no	no	no	I	M	<1%	0
P40	56	F	SB (frontal)	no	no	no	I	F	<1%	0
P41	56	M	convexity (P)	no	no	no	II*		<5%	4<M<20
P42	56	F	convexity (T)	no	no	no	I	M	3<M<5%	
P43	74	F	SB (frontal)	no	no	yes	I	M		0
P44	47	F	SB (frontal)	no	no	no	I	T		0
P45	62	F	SB (temporal base)	no	no	no	I	M	<3%	
P46	55	M	convexity (fontal)	no	no	no	I	M	1%	0
P47	32	F	tentorium	no	no	no	I	T	<3%	0
P48	56	F	SB (F)	no	no	no	I	M		0
P49	65	F	SB (T)	no	no	no	II*	M	2<M<5%	4<M<20
P50	58	F	convexity (F)	no	no	no	II*	F	<3%	4<M<20
P51	47	F	convexity (F)	no	no	no	II*		2<M<5%	4
P52	52	F	convexity (P)	no	no	no	I	T	<3%	0
P53	69	M	convexity (PF)	no	no	no	I	F		0
P54	32	M	convexity (F)	no	no	no	I	F	<1%	0
P55	57	M	convexity (F)	no	no	no	II*		<7%	4<M<20
P56	59	F	SB	no	no	no	I	T		0
P57	76	F	convexity (P)	no	no	no	II*	M	2<M<5%	<4
P58	70	F	SB (temporal base)	no	no	no	I	P		0
P59	47	M	convexity (F)	no	no	no	I	Mi		0
P60	54	M	SB (PF)	no	no	no	I	M		0
P61	52	F	convexity (F)	no	no	no	I	M	<1%	0
P62	35	M	convexity (T)	no	no	no	I	M		0
P63	67	F	SB (frontal)	no	no	no	I	M	<1%	0
P64	66	F	ventricular	no	no	no	II	M	20%	<4

Location (F = frontal, T = temporal, P = parietal, PF = posterior fossa, SB = skull base); infiltrated tissues (nd = no identification possible on pathological examination as the tumor removal concerned its extension outside the invaded sinus; hence infiltration was stated on MRI or CT data only; sup longitudinal sinus = superior longitudinal sinus); WHO grade (II* = grade II according to the number of mitoses per field (Perry1999)^11^; pathology subtype (M = meningothelial, Mi = microcystic, T = transitional, P = psammomatous, S = secretory).

The study was approved by the Biological Resource Center Ethics Review Board at the Grenoble University Hospital. Written consent was obtained from each patient or the patient's family.

### SELDI-TOF Mass Spectrometry

Cryostat slices (10 µm thick) were suspended in 300 µL of lysis buffer (Reporting Lysis Buffer, Promega, Madison, WI, USA) containing a mix of protease inhibitors (Boehringer/Roche, Meylan, France), to obtain a final protein concentration close to 2 µg/µL. After 30 min incubation on ice and centrifugation (10,000 *g* for 10 min at 4°C), supernatant was diluted in a binding buffer (100 mM Tris and 0.1% TritonX100 at pH 8.0) to a final protein concentration of 0.1 µg/µL, and 100 µL of this suspension was applied to Q10 anion-exchange active binding surfaces of SELDI ProteinChip Arrays (Bio-Rad, Marnes-La-Coquette, France). The chips were washed three times with the binding buffer, then once with the binding buffer without Triton for 5 min, then once with 2 mM HEPES at pH 7.5. For other analysis purposes, protein fractions were analyzed on the hydrophilic NP20 ProteinChip Arrays. In this case, active surfaces were washed with water only. Sinapinic acid was used as the ionization matrix and ProteinChip Arrays were analyzed both for low- and high-mass range optimization in a Bio-Rad PCS4000 mass spectrometer. Protocols for preparing the samples and the arrays for analyses were followed rigorously since quantitative measurements of proteins by laser-desorption ionization mass spectrometry depend on critical parameters such as variability in matrix crystallization and therefore ionization efficiency. In addition, all spectra were calibrated using Bio-Rad all-in-one protein calibrants.

### Data Analysis

Peaks were automatically detected and normalized to total ion current intensity in the 4- to 100-kDa range using the Biomarker Wizard software (Ciphergen, Fremont CA, USA). Peak information outputs were used for unsupervised biomarker clustering. Unsupervised clustering relies on methods that can mine through data, extracting relevant information, independently of the information regarding the invasive or noninvasive phenotypes of the tumors. This was computed by hierarchical clustering using Cluster software. To investigate the correlation between protein peaks, visualization was performed with the TreeView Software (software packages available at http://dnagarden.ims.u-tokyo.ac.jp/en/doku.php) [13].

For supervised clustering, samples from the two phenotypic groups (infiltrative/invasive and noninfiltrative/noninvasive according to histopathological criteria) were allocated to equivalent training and testing sets without preset criteria (Table 2). Only the training set samples were classified for training the Biomarker Pattern Software 5.0.2 (Ciphergen). Then the sensitivity and specificity parameters were assessed, with the same software after analysis of the testing set data.

**Table 2:** Features of the sample groups.

	Training set	Testing set	Total
Number of patients	32	32	64
Age: mean in years (range)	58.4 (32–79)	57.2 (35–76)	(32–79)
Men/women	6/26	7/25	13/51
Total number of noninfiltrative tumors	15	15	30
Total number of tumors with infiltrative or invasive features (tumors may have several infiltrative features)	17	17	34
Cavernous sinus or sagittal sinus infiltration	12	11	23
Bone infiltration	13	10	23
Cortex invasion	1	1	2

### Purification and Identification of the Protein Marker

The 53-kDa biomarker was purified by chromatography through a Q HyperD anion exchange column (Pall Biosepra, Cergy Saint Christophe, France) as follows. A lysis supernatant obtained as described above was loaded on the top of a 0.5-mL Q column equilibrated in the binding buffer. The column was then washed by a solution containing 5% acetonitrile, 0.5% TFA, and 50% isopropanol. Fractions were collected during elution with a solution made of 16% acetonitrile, 0.1% TFA, and 33% isopropanol. Aliquots were checked by mass spectrometry analyses with NP20 ProteinChip Arrays.

Fractions containing the purified biomarker were pooled and submitted to a 1-D electrophoresis migration under denaturing conditions in 15% polyacrylamide gels, according to standard procedures. After Coomassie blue staining, the 53-kDa band was cut and subjected to GluC endoproteinase or trypsin proteolysis as described [14]. The peptide mixtures were bound to NP20 ProteinChip Arrays and analyzed using SELDI-TOF mass spectrometry for fingerprinting analysis and with a MALDI-TOF/TOF (ABI 4800) mass spectrometer to identify specific peptides. Trypsin digests were also analyzed by nano-LC-MS/MS with the nanochromatographic system (Ultimate 3000 – Dionex; Sunnyvale, CA, USA) directly coupled to an LTQ-Orbitrap (Thermo Fischer Scientific; Bremen, Germany). Data were collected and processed automatically using Masslynx 3.5 software. Protein searches were performed in the SwissProt-TrEMBL decoy database (http://www.expasy.org/sprot/) using the Mascot program (Matrix Science; http://www.matrixscience.com).

Fractions containing the purified biomarker were vacuum dried. The sample was then resuspended in Tris-Cl 0.1 M, MgCl2 10 mM, pH 8.8, and the pH of the suspension was controlled. Then phosphatase treatment was performed for 1 h at 30°C with calf alkaline phosphatase. Two different types of phosphatase were used: soluble (Roche) or agarose-crosslinked (Sigma; St Louis, MO, USA) calf intestine alkaline phosphatases.

Both the purified protein and the phosphatase-treated sample were analyzed by SELDI-TOF mass spectrometry using NP20 ProteinChip Arrays or Q10 anion-exchange ProteinChip Arrays as described above. Controls that were run in the absence of phosphatase established that the observed mass shifts were not due to any protease activity.

## Results

### Intratumoral Protein Content Analysis by SELDI-TOF Mass Spectrometry

Solubilized protein extracts were prepared from the tissue samples of 64 meningiomas including 32 infiltrative, two brain-invasive, and 30 noninfiltrative tumors (Tables 1 and 2). Protein patterns were generated by SELDI-TOF mass spectrometry with the Q10 anion-exchange ProteinChip Arrays. Mass spectra detected 69 different statistically significant peaks (in the 4- to 100-kDa range) that were putative biomarkers distinguishing the noninfiltrative meningiomas from the others.

**Figure 1:**
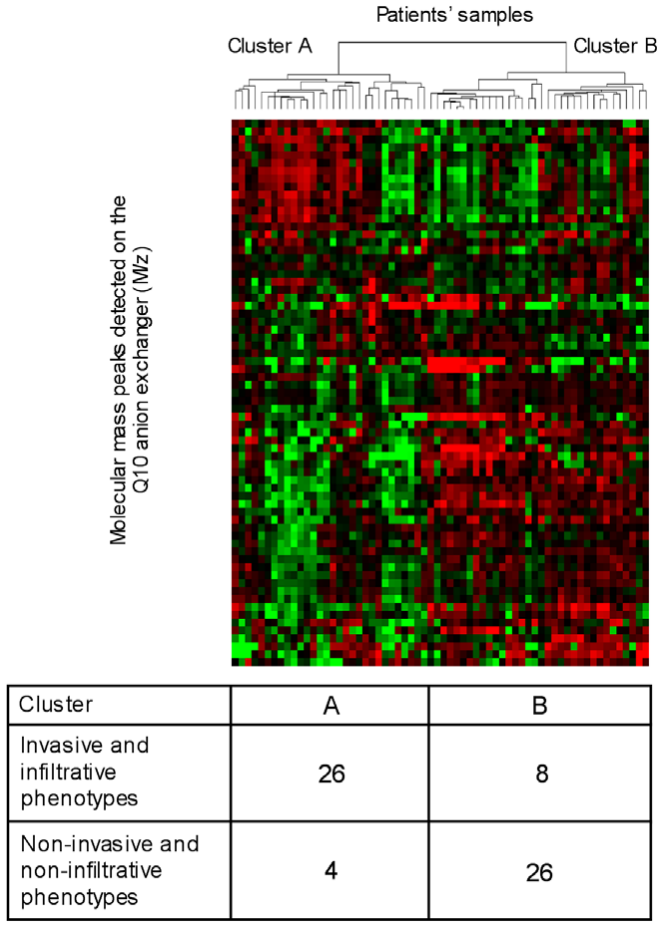
Unsupervised hierarchical clustering of the tumors. Two-dimensional hierarchical clustering of 64 tumors (32 infiltrative, two invasive, and 30 noninvasive tumors) was performed with 69 molecular mass peaks after SELDI-TOF mass spectra processing with the Biomarker Wizard software (Ciphergen). Candidate markers and patient samples were clustered using complete linkage clustering methods from Eisen's cluster software. Clustered trees are displayed using Eisen's Treeview software. Red squares denote high marker concentration in comparison to average; green squares denote low concentration in comparison to average. The numbers of infiltrative or invasive samples and of noninfiltrative or noninvasive samples in the two clusters are indicated.

We performed a hierarchical clustering analysis based on the whole set of protein peaks detected by the Biomarker Wizard software using the Cluster and TreeView programs. This unsupervised clustering segregated most of the infiltrative/invasive and noninfiltrative tumors into two distinct clusters (Figure 1). The accuracy of this classification was about 82% (four noninfiltrative and eight infiltrative/invasive samples were misclassified to the two opposite groups). The result of this clustering suggested that an infiltrative/invasive versus noninfiltrative phenotypic segregation of the tumors could be possible based on the SELDI-TOF mass spectrometry patterns of the tissue extracts.

### One Marker Adequately Differentiates Infiltrative/Invasive and Noninfiltrative Tumors

**Figure 2:**
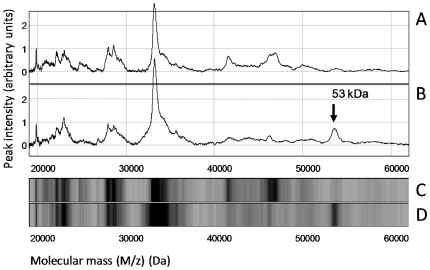
Protein profiling by SELDI-TOF mass spectrometry on Q10 anion-exchange ProteinChip Arrays. Two representative sample extracts were analyzed, one from an infiltrative tumor (A and C) and one from a noninfiltrative tumor (B and D). (A and B) Mass spectra traces in the 20- to 60-kDa range. (C and D) gel views of the A and B spectra, respectively. Arrow in panel B shows the 53-kDa marker in the noninfiltrative tumor extract.

We then attempted to recognize the smallest set of markers with the best discriminative ability to appropriately classify the samples. Briefly, a training set of samples composed of 17 infiltrative/invasive and 15 noninfiltrative tumors was designed (Table 2) and a supervised clustering was done using the Biomarker Pattern Software. This analysis revealed that a single, unique molecular marker is adequate for discrimination of the noninfiltrative versus infiltrative/invasive tumors. After clustering the training set, this information was used for predictive classification of the samples in the testing set (Table 2). Sensitivity and specificity values of 86.7% and 100%, respectively, were obtained for this marker. This emphasized that noninfiltrative tumors behave as a homogeneous group of tumors that can be easily distinguished from infiltrative/invasive tumors on the basis of the detection of this newly discovered marker. In addition, tumors with infiltrative or invasive phenotypes shared similar profiles with a very low detection level of the marker, independently of their individual infiltrative features (i.e., individual or combined infiltrations/invasions of sinuses, bone, or brain). This marker was characterized by a 53-kDa molecular mass. A typical comparison of the mass spectra recorded in the range between 20 and 60 kDa for two tumors (one noninfiltrative tumor and one bone-infiltrative tumor) is shown in Figure 2.

**Figure 3:**
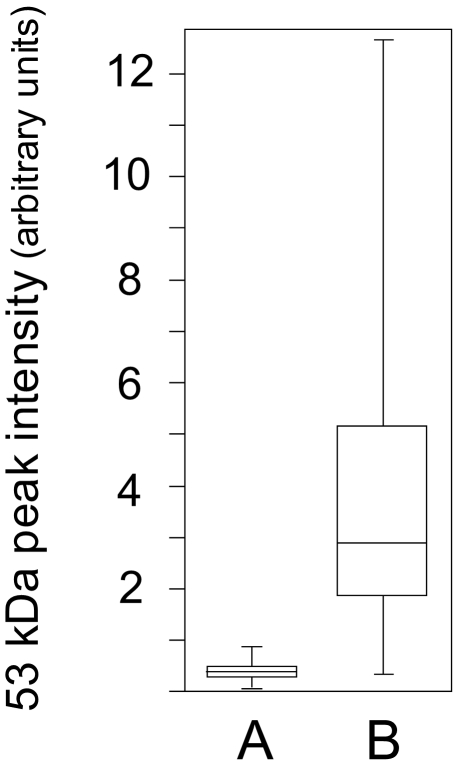
Quantitative assessment of the 53-kDa marker in the clinical samples. Tumor extracts were analyzed using SELDI-TOF on Q10 anion-exchange ProteinChip Arrays, as shown in Figure 2. Signal intensity at the 53-kDa mass level were measured and calculated parameters plotted for (A) infiltrative/invasive tumor samples, (B) noninfiltrative tumor samples. Medians, 25^th^ and 75^th^ percentiles are marked with line segments across the boxes and the lowest and highest signal values with bars.

Under the experimental conditions used herein, the intensity of the 53-kDa molecular mass was found to average at a value of 0.35 arbitrary units (median, 0.33) for the extracts of the infiltrative/invasive tumors, contrasting with the value of 3.94 arbitrary units (median, 2.90) measured for the samples from the noninfiltrative tumors with a highly discriminative significance level (*p*-value<0.0001) (Figure 3).

### Identification of the 53-kDa Biomarker

**Figure 4:**
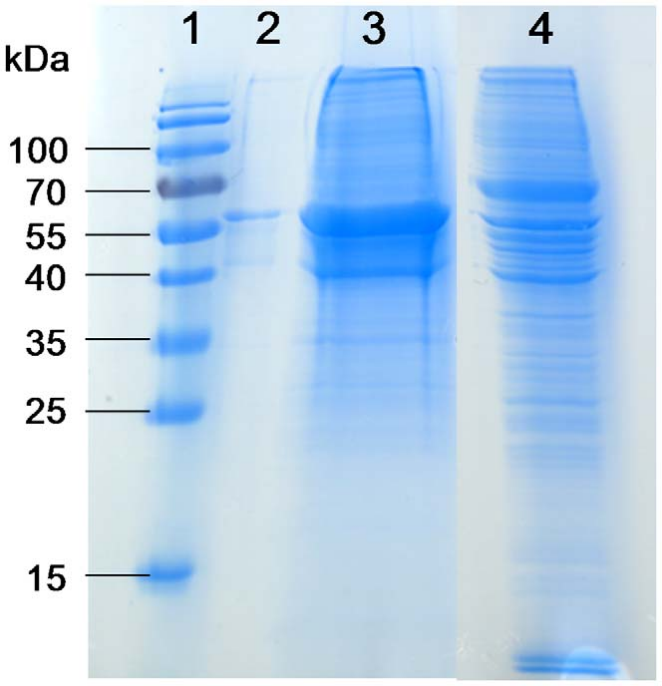
Purification of the 53-kDa marker. Lysates from noninfiltrative tissues were submitted to the purification method reported in Materials and Methods. Quality control was run on a 12% polyacrylamide gel under denaturing conditions and the 53-kDa band was cut and further subjected to analysis for identification. Lane 1: mass ladder; lanes 2 and 3: purified 53-kDa marker (0.5 µg and 20 µg, respectively); lane 4: meningioma lysate.

In the first step, the optimal conditions for binding the marker to anion-exchange active surfaces and for elution after binding were determined on a miniaturized scale. This was done by incubating solubilized tissue extracts in the presence of various buffers on Q10 ProteinChip Arrays and assaying by mass spectrometry the desorption of the bound biomarker after incubation with several eluting solutions. Large-scale purification of the biomarker was then carried out on Q HyperD columns (the chemical properties of the column resin are identical to those of the Q10 active surfaces) using the binding and eluting solutions specified previously. Purification was completed by 1-D polyacrylamide gel electrophoresis under denaturing conditions (Figure 4) and the 53-kDa stained band was excised and submitted to digestion by proteolytic enzymes.

**Figure 5:**
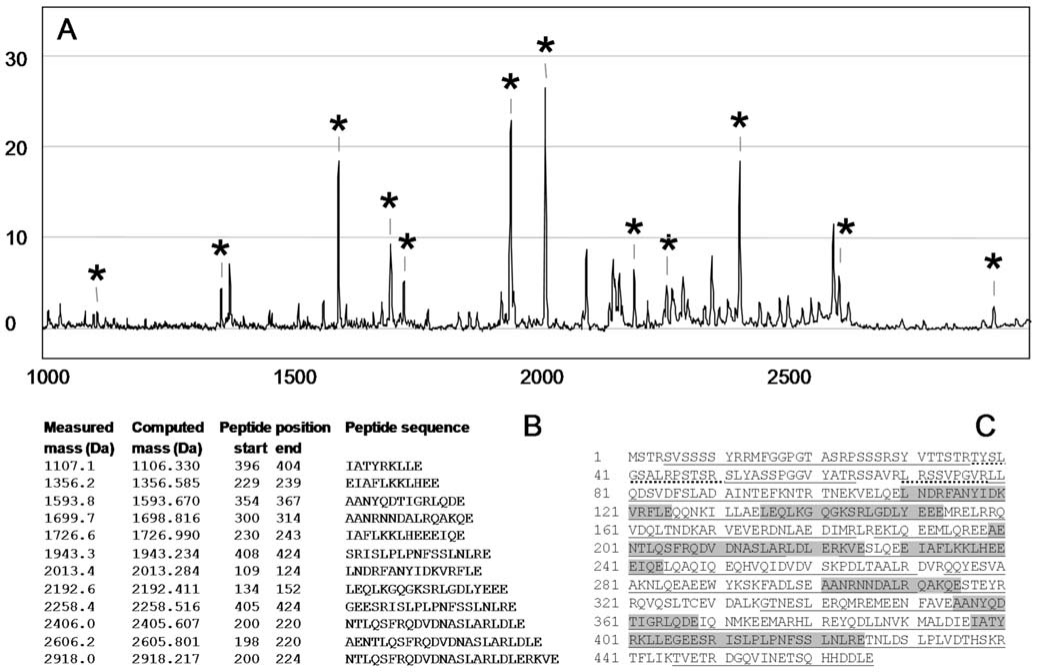
The 53-kDa marker identified as vimentin. After purification by chromatography and electrophoresis, the 53-kDa marker was cleaved by proteases. (A) peptide fingerprint after GluC endoproteinase digestion obtained by SELDI-TOF analysis on NP20 ProteinChip Arrays. Peptides with measured molecular masses matching those of the computed GluC endoproteinase proteolytic peptides of vimentin are indicated with an asterisk. (B) GluC endoproteinase peptides of vimentin identified by SELDI-TOF are listed according to their masses. (C) Mapping of the peptides identified by either peptide mass fingerprinting or nanoLC-MS/MS to the vimentin sequence. Sequences highlighted in grey correspond to GluC endoproteinase peptides identified in B. Underlined sequences correspond to 60 trypsin peptides identified by nanoLC-MS/MS. Phosphorylated peptides (37-50 amino acid and 70-78 amino acid peptides) are underlined with dots.

After this treatment, peptides released from the piece of gel were analyzed by SELDI-TOF mass spectrometry to generate a peptide mass fingerprint and by nanoLC-MS/MS to obtain additional information regarding the amino-acid sequence of the peptides. Both approaches demonstrated that the purified 53-kDa biomarker was vimentin. A peptide profile obtained after GluC endoproteinase digestion of the 53-kDa biomarker (cleavage at the carboxyl groups of glutamic acid residues) is shown in Figure 5A. On the basis of the molecular masses, twelve peaks in this profile were found to match with GluC endoproteinase fragments of vimentin (Figure 5B and 5C). In addition, after trypsin treatment of the purified biomarker (cleavage at the carboxyl groups of arginine and lysine amino acids) and peptide analysis using nanoLC-MS/MS, data were analyzed against the Swiss-prot TrEMBL decoy database. The intensities and continuity of matched fragment ions in b, y series led to unambiguous identification of 60 peptides of vimentin with high scores obtained through Mascot search engines. The cumulated length of all the trypsin peptides identified encompassed 91% of the total length of vimentin (Figure 5C).

We then immunodetected vimentin in meningioma tumor slices with a vimentin-directed monoclonal antibody. No significant quantitative correlation was observed for the different tissue samples, between antigen reactions and their infiltrative/invasive or noninfiltrative status (not shown). Assessment of the total concentration of vimentin by SELDI-TOF analyses on NP20 ProteinChip Arrays to allow detection of both natural and modified forms of vimentin also failed to detect any significantly different concentration in the samples. Moreover, we found no significant variation of the vimentin-transcript levels in the various samples when assayed by cDNA hybridization to oligonucleotide-microarrays (not shown). This indicated that assay of the vimentin transcript in these tumors cannot be used to predict invasiveness. All these observations were taken as indications that in noninfiltrative tissues, the vimentin detected by adsorption to the Q10 anion-exchange ProteinChip Arrays was in fact a non-native form of vimentin.

**Figure 6:**
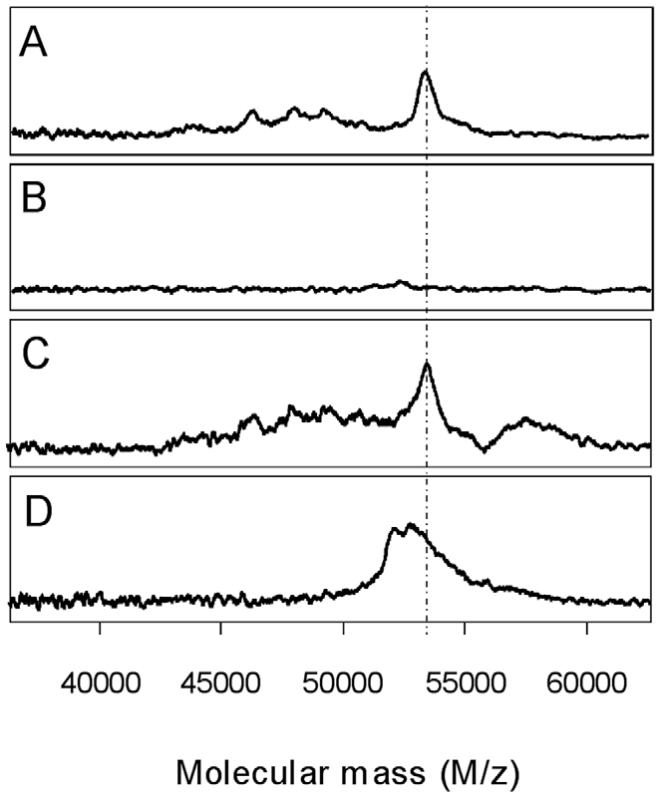
The 53-kDa marker is a phosphorylated form of vimentin. Purified 53-kDa marker from noninfiltrative tissue extracts was treated by alkaline phosphatase and samples were analyzed using SELDI-TOF MS. Analyses were performed on Q10 anion-exchange ProteinChip Arrays (A and B) or hydrophilic NP20 ProteinChip Arrays (C and D). (A and C) Controls with untreated purified 53-kDa marker. (B and D) phosphatase-treated marker.

We were intrigued by the tight binding of vimentin on the Q10 anion-exchange ProteinChip Arrays. The protein was not eluted even by a pH 3.0 buffer, even though vimentin has a computed isoelectric point of 5. It was hypothesized that the presence of phospho-esterified amino acids in the protein would explain this striking anionic feature. This was ascertained by the fact that after alkaline phosphatase treatment, the form of vimentin, purified from noninfiltrative tumors, was no longer able to bind to the Q10 anion-exchange ProteinChip Arrays (Figure 6). In this dephosphorylated state, vimentin was still able to bind to the hydrophilic NP20 ProteinChip Arrays but exhibited a molecular mass that was lowered by about 300 Da. This mass shift suggested that nearly three phospho-groups per molecule of modified vimentin were removed during the phosphatase treatment. NanoLC-MS/MS data analysis indicated that two vimentin tryptic peptides exhibited a mass supplement corresponding to the addition of one phosphate group (peptide from amino acid 37 to 50, which contains four serine, two threonine, and one tyrosine residue, and peptide from amino acid 70 to 78, which contains two serine residues). Commercially available polyclonal antibodies directed against seven different phosphorylated-vimentin peptides were used in Western blots to identify specific vimentin phosphosites in lysates from noninfiltrative or infiltrative meningiomas. Phosphorylated epitopes were detected in the meningioma samples by only two of these antibodies. However, the intensities of the immunoreactivities were found to be identical in both types of lysates. This result suggests that at least serine residues 51 and 72 (see vimentin sequence in Figure 5) are not specifically phosphorylated in the noninfiltrative meningiomas. Additional investigations are therefore required to identify the specific phosphorylated amino acid residues in the vimentin form that is present in greater amount in noninfiltrative meningiomas.

Finally, this study demonstrated that in meningiomas, an increase of vimentin concentration is probably not a molecular event indicative of invasiveness per se but more accurately the presence of the protein in a multiphosphorylated form is an indicator for noninvasiveness.

## Discussion

### Phosphorylated Vimentin as a Marker for Differentiation between Infiltrative/Invasive and Noninfiltrative Meningiomas

The proteomic patterns of meningiomas were analyzed with the aim of discovering new biomarkers or molecular events that are indicative of their infiltrative or invasive phenotypes. These phenotypes were defined as the feature of meningiomas that infiltrate adjacent tissues such as bone or venous complex sinuses (cavernous sinus or superior longitudinal or lateral/sigmoid sinuses) or invade brain tissue. Meningiomas allowed a molecular characterization of proteomic markers related to infiltration/invasion, in the clinical context of low-grade, low-proliferation benign tumors. This proteomic study was conducted using a SELDI-TOF mass spectrometry approach on solubilized tissue extracts. We discovered one biomarker that has a clear ability to discriminate between infiltrative/invasive and noninfiltrative tumors. This marker was identified as a multiphosphorylated form of vimentin. This post-translational modified vimentin is found in noninfiltrative tumors at easily-detectable levels.

### Vimentin in Physiological and Pathological Contexts

Vimentin is a 466-amino-acid-long protein with a 53,652-Da molecular mass (Uniprot, http://www.uniprot.org, P08670). It is encoded by a single-copy gene located on chromosome 10q13. Vimentin is naturally expressed in nonepithelial cells and in mesenchymatous cells. In the central nervous system, vimentin is absent from oligodendrocytes, mature astrocytes, and neurones, but it is expressed in the Schwann cells and ependyma cells. Under certain specific physiological or pathological conditions, cellular levels of vimentin might be up- or downregulated. Vimentin was found to be detectable in astrocytomas and glioblastomas (grade IV astrocytomas), meningiomas, and ependymomas but not in oligodendrogliomas and medulloblastomas [15], [16].

Numerous data in the literature have already reported that some cytoskeleton constituents are expressed in epithelial cells with concomitant acquisition of new morphological and migratory phenotypes, a phenomenon called epithelial-mesenchymal transition. More specifically, overexpression of vimentin, one polypeptide of the type III intermediate filaments, is often believed to be correlated with the invasiveness or increased metastatic potential of many tumors [17]–[20]. Consequently, detection of vimentin in tumors has been considered a biological marker of poor prognosis [21]. However, several other authors disagreed with this statement [22]–[24]. Additionally, overexpression of vimentin induced by stable transfection of expression constructs in tumoral cells was unable to enhance invasiveness in poorly invasive prostatic tumor [25] or was shown, on the contrary, to decrease hepatocarcinoma proliferation [26]. Thus, understanding the correlation between overexpression of vimentin and tumor invasiveness still calls for additional studies.

Vimentin is a target for several post-translational modifications and some of these modifications may be linked with diverse pathologies. For example, upregulation of an N-terminus truncated vimentin was observed in Ha-ras transfected tumorigenic rat liver cells [27]. In addition, an unidentified specific vimentin isoform was found to elicit antibody production in pancreatic cancer [28]. In rheumatoid arthritis, modified citrullinated vimentin isoforms are generated and act as autoantigens [29], [30]. Antibodies against citrullinated proteins are assumed to play a role in the pathogenesis of arthritis [31]. Finally, O-linked beta-N-acetylglucosamine modification of vimentin can be observed during cell cycle progression [32].

Vimentin is also a well-known substrate for several kinases. Close to 40 different phosphorylated sites have been experimentally identified (Uniprot, http://www.uniprot.org, P08670 and references therein) [33]–[38]. Roughly 30 serine residues are targets for phosphorylation and 21 of them are located in the first 100 amino acids at the N terminus of vimentin. Five threonines and four tyrosines were also identified as phosphosites (Uniprot, http://www.uniprot.org, P08670 and references therein). Phosphorylation has been reported to be catalyzed by numerous kinases such as cAMP-dependent protein kinase (PKA), protein kinase C, Ca^++^/calmodulin-dependent protein kinase-II, cdc2 kinase, PKN, Rho-kinase, mitogen-activated protein kinase-activated protein kinase-2 (MAPKAP kinase-2), and p21-activated kinase (PAK) [33]–[40]. It is also worth mentioning that vimentin is one of the usual protein markers detected by immunohistochemistry for the definite diagnosis of meningiomas whatever their invasive or noninvasive phenotype is. Our present data clearly illustrate that, in meningiomas that infiltrate bone or sinuses, the infiltrative behavior of these tumors correlates better with the level of phosphorylated vimentin than with the global expression level of this protein. With a structural function of vimentin in the cytoskeletal scaffolding, it can be assumed that vimentin plays a key role in the process of tumor cell migration or motility. It is interesting to note that in breast tumor cells, vimentin filament assembly plays a direct role in the stability of a type of cellular protrusions, called tubulin microtentacles, that are involved in tumor invasiveness [41]. A decrease in microtentacle frequency was observed in noninvasive, non-vimentin-expressing breast carcinomas or in cells expressing a dominant-negative vimentin mutant that promotes vimentin filament disruption or after cell treatment with inhibitors of PP1/PP2A phosphatases. This last observation is in line with the fact that vimentin phosphorylation is known to play a role in disassembly of the intermediate filaments during mitosis [32], [42]. Accordingly, it can be hypothesized that excessive phosphorylation of vimentin, as a process leading to vimentin filament disassembly, may underlie important steps in migratory control of meningioma cells.
